# Thesis on the Endemic Bronchocele of Nassau and the Electorate of Hesse

**Published:** 1845-07

**Authors:** 


### Art. XVI.

*De Thyrophymate Endemico per Nassoviam et Hessiam Electoralem.* Dissertatio inaug. med. quam scripsit Carolus Philippus Falck.—*Marburg,* 1843.

*Thesis on the Endemic Bronchocele of Nassau and the Electorate of Hesse.* By C. P. Falck.—*Marburg,* 1843.

The existence of bronchocele as an endemic disease in Nassau and Hesse appears hitherto to have attracted little attention, the only authors quoted by Dr. Falck being Drs. Mombert* and Fleisch,† who severally described the disease as they observed it in the district of Wanfried on the Werra, and in the village of Nentershausen. The facts, however, collated by the author show that bronchocele is a frequent disease among the inhabitants of most portions of the two provinces, and in certain districts is endemic, to an extent scarcely less than in the valleys of Switzerland.

The data on which Dr. Falck has founded his estimate of the comparative prevalence of the disease, are deduced from the returns of the conscripts levied for the armies of the two principalities between the years 1831 and 1840, and 1836 and 1842 respectively. From these he has furnished tables of the number of men selected from each district and village annually; together with those who were found to labour under bronchocele so as to unfit them for service, or require their treatment previous to enrolment. The proportion which the goitrous bear to the whole of the conscripts being thus ascertained, he is enabled to estimate with considerable exactitude the number of persons in the general population of the several districts and villages, who probably labour under the disease, and to investigate the causes conducing to its development in each.

The following is an example of the mode of investigation adopted: in the bailiwick of Braubach, in the southern province of Nassau, the number of conscripts levied annually during the ten years were respectively 142, 131, 114, 126, 141, 140, 147, 130, 167, and 164, in all 1402, or a mean of 140·2 annually; and of these 37, or on the average 3·7, each year were found to be affected with bronchocele; the proportion of those labouring under the disease being to the whole of the conscripts as 1 to 37. Of the total population of this district, 11,092 in number, the proportion of those affected with goitre has been therefore estimated at 232. The population is distributed in 19 villages, and the conscripts levied from 13 of these numbered a larger or smaller proportion of the goitrous: thus in the village of Fachbach, containing a population of 411 inhabitants, 5 conscripts are selected annually (1 to 80 inhabitants,) or during the 10 years 50; and of these 7, or more than $\frac{1}{7}$th, were found to labour under bronchocele; and consequently it has been inferred that the disease is endemic in this village. The lengthened period over which the returns extend obviates the fallacy which might be occasioned by a casual occurrence of the disease, and in many instances the rejections of goitrous persons are found to recur year after year; thus in Weilburg, in the eastern portion of the southern province of Nassau, goitrous persons are returned in 8 out of the 10 years. The greater liability of females to the affection, which when the

* Hufeland's Journ. B. 77. Sept. 1833, p. 90.

† Handbuch über die Krankheiten der Kinder, etc. 3. B. Leipsig, 1807, p. 388.

disease is of less frequent occurrence, certainly obtains, (as in England,) does not, from Dr. Falck's observation at Ockershausen in Hesse, appear to exist in those districts. He has indeed satisfied himself of the general correctness of his mode of investigation; though the returns must be regarded as indicating a prevalence of the disease less than that which really exists; rejections of goitrous persons often taking place in consequence of their being below the required standard, or on account of some other malady with which the disease is conjoined; and thus it occasionally happens that in villages in which the disease is known to be endemic no persons are returned as rejected on this ground.

I. *The endemic bronchocele of the duchy of Nassau.* The duchy of Nassau, situated on the left bank of the Rhine, is divided into two provinces by the river Layne. In both of these the country consists of elevated plains intersected by deep valleys and mountains. The southern portion or Taunus is situated between the valleys of the Layne and Mayne; its highest peak the "Grosser Feldberg" attains a height of 2721′:* this mountain range descends abruptly towards the valley of the Mayne on the south, while to the west and north it sinks more gradually towards the Rhine and Layne.

The northern portion or Westerwald rises gradually from the valley of the Layne, forming an elevated range of which the Salzberge Kopf is the highest peak: 2600 feet to the north these mountains become continuous with those of the Lower Rhine; to the west they slope to the valley of the Rhine; and both provinces border on the fertile district of Wetterau on the east.

Geologically these provinces consist chiefly of the transition strata, commencing with the grauwacke and argillaceous schistus, which in Westerwald are interspersed with basalt and other rocks of igneous origin. In Taunus the argillaceous schistus gives place to clay and sand in the valley of the Mayne. In the upper course of the Layne, spilite and greenstone occur.

The valleys are freely irrigated by streams. The climate is tempestuous in the mountains, mild and agreeable in the valleys.

Of the total number of conscripts levied in those provinces between the years 1831 and 1840 inclusive, 50,913 in number, 514 were found to labour under bronchocele, or 1 in 99 nearly, and thus out of the total population of the duchy, amounting to 393,710, Dr. Falck estimates that 4026 are goitrous. Of the conscripts 25,736 are drawn from the southern province, and of these 271 were affected with bronchocele; and 25,177, of whom 243 were goitrous, were levied in the northern province: the proportion affected being thus somewhat greater in the latter district. The disease appears to prevail to very varied extent, not only in the several districts of each province, but also in the villages included in each district: thus in the bailiwicks of Braubach, Weilburg, and Runkel, the proportion which the goitrous bore to the whole of the conscripts levied was respectively 1 in 37, 1 in 54, and 1 in 60; while in Rennerod and Hachenburg it amounted to only 1 in 378 and 1 in 516, and in Marienberg and Reichelsheim no rejections on the ground of the persons labouring under bronchocele, were returned.

* This accent-mark of extent is alone used in the original: does it indicate *feet*?

Our space will not permit our presenting more than an outline of the general results obtained by Dr. Falck relative to the character of the strata and site of the 59 villages, in which he is led to infer that bronchocele exists either as a frequent or endemic disease.

*Character of strata :*

| | |
|---|---|
| Situated in Grauwacke and clay slate | 34 |
| ,, Argillaceous schistus | 8 |
| ,, Spilite | 7 |
| ,, Limestone—Transition ? | 4 |
| ,, Greenstone | 4 |
| ,, Magnesian Limestone | 1 |
| ,, Basalt | 1 |
| ,, Clay and sand | 1 |

*Site.* The sites of 52 of the villages are given as follows:

| | |
|---|---|
| Situated on the summits of hills | 6 |
| ,, Northern slope | 5 |
| ,, Eastern ,, | 14 |
| ,, Southern ,, | 9 |
| ,, Western ,, | 10 |
| ,, a slope having a North-west aspect | 3 |
| ,, ,, North-east ,, | 1 |
| ,, ,, South-east ,, | 2 |
| ,, ,, South-west ,, | 2 |

The direction of the valleys are:

| | |
|---|---|
| From North to South | 15 |
| South to North | 5 |
| East to West | 5 |
| West to East | 6 |
| North-east to South-west | 4 |
| North-west to South-west | 3 |
| South-east to North-west | 2 |

Two of the villages are situated in valleys nearly surrounded by mountains, and so are built on elevated sites. Lastly, the prevalence of bronchocele does not correspond with the extent either of the forests, meadows, or stagnant waters.

II. *The endemic bronchocele of the electorate of Hesse.* The electorate of Hesse consists of four provinces: 1, Lower Hesse, to the north, containing Cassel, the capital, together with the districts bordering on the Weser, and its tributaries the Werra, Fulda, and Diemal, and the separate county of Shaumburg; 2, Upper Hesse, to the north-east, bordering on Nassau, and containing the circles of Marburg, Kirchenhain, Frankenberg, and Ziegenhain; 3, Buchonia, to the south, containing the circles of Fulda, Hünefeld, Hersfeld, and the separate lordship of Smalkalden; 4, Hanau, to the south-east, containing Hanau, Gelnhausen, and Schlüchtern. Of these provinces, the Lower (with the exception of the county of Shaumburg), a portion of the Upper, and Buchonia consist of a series of valleys and ranges of hills, which in some places attain a considerable elevation, as the Habichtswald (1312′), between the rivers Elder and Diemal; the Rheinhard, between the latter river and the Werra; the Sillingswald, and the Meisner (2200′), between the Werra and Fulda; and the Knüll (1156′), between the Fulda and Schwalm. The lordship of Smalkalden is situated within the range of the Thuringewald, and contains the Inselberg (2855′), one of its loftiest peaks. The province of Buchonia forms a plain elevated 800′, and situated between the ranges of the Rhoenwald and the Vogelsberg; and Hanau is situated between the latter range and the Forest of Spessart.

Hesse is situated principally on the (new?) red sandstone formation, beneath which the magnesian limestone occurs in some places, as in the vicinity of Bieber and Reichelsdorp; and in others, as around Frankenberg, a cupreous schistus, and the new red conglomerate, and grauwacke. The red sandstone is covered by a shell limestone, or molassi, from which flow saline springs. In the districts of the Rhoenwald and Vogelsberg, the Meisner, and around Kirchenhain and Zeigenhain, the tertiary strata are interrupted by basalt. In Smalkalden micaceous schistus, gneis, and granite occur in large tracts.

The mean temperature of this district may be stated at 8·9 centigrade, which is 1·1 less than that of Baden (48° Fahrenheit).

Nearly one third of the electorate is covered by forest: the proportion being much less than this in the Lower and Upper Provinces, and amounting to upwards of one half in Smalkalden—the trees belong chiefly to the oak genus in the former provinces, and in Smalkalden to the coniferæ.

The inhabitants are employed in agriculture, the tending of cattle, and in the forests; they chiefly subsist on potatoes, and in some parts, as around the Schwalm and in Hanau, on fruits. Sobriety does not appear to be one of their virtues, much corn spirit being drunk in the Lower Provinces, in Buchonia ale, and in Hanau cider and wine. They possess robust but not elegant forms. They suffer much from hemorrhoids, and in some of the valleys from scrofulous affections and intermittent fevers, more especially in the confined valley of the Ohm (Amanal), and in those of the Mayne and Weser, where basalt occurs. In Cassel, Hofgeismar, and Witzenhausen, varicocele is common amongst the conscripts; in Wolfhagen, Fritzlar and Marburg, valgus; and in Smalkalden, Eschewege and Rotenburg, bronchocele. From the returns of the conscripts levied in the electorate between the years 1837 and 1842 inclusive, it appears that of the total number of 49,916, 1071, or 1 in 47, were found to labour under bronchocele, the proportions in the several provinces being as follows:

| | | | | |
|---|---|---|---|---|
| Lower Hesse, | conscripts levied . | 19,823 | goitrous . | 402—1 in 49 |
| Upper Hesse | ,, | 9149 | ,, | 927—1 in 94 |
| Buchonia | ,, | 10,931 | ,, | 405—1 in 27 |
| Hanau | ,, | 10,010 | ,, | 167—1 in 60 |

Of the total population, amounting to 712,318, Dr. Falck estimates that 15,195 labour under bronchocele. The disease seems to be more generally prevalent in Hesse than in Nassau, in each district a larger or smaller number of rejections having taken place, and, in those most affected, the proportion of the population labouring under the disease being greater. In Smalkalden, Eschwege, Rotenburg, and Witzenhausen the proportion of the conscripts found to be goitrous was as high as 1 in 11, 1 in 24, 1 in 27, and 1 in 28. In Shaumburg and Kirchenhain, on the contrary, it amounts to only 1 in 305 and 1 in 383.

Of the whole of the towns and villages the disease appears to be of frequent occurrence or endemic in 93. The geological situations of these villages are as follows:

| | |
|---|---|
| On Magnesian and shell limestone | 84 |
| Primitive rocks | 3 |
| Clay and sand | 3 |
| Molasse | 2 |
| Volcanic rocks | 1 |

Dr. Falck remarks, that both in Nassau and Hesse the disease does not occur frequently in places situated on strata of volcanic origin. In Hofgeismar and Wolfhagen, also, it is rarely met with; throughout these districts the shell limestone abounds. In Smalkalden where the disease is most frequent, besides the situation of the villages on the magnesian limestone, some influence may perhaps be exerted by the extensive pine forests. Though the disease, as will be seen by the numbers given, is less frequent in the upper than in either of the other provinces of Hesse, it is, on the contrary, very prevalent in certain portions of that district, this is particularly the case in the village of Ockershausen in the circle of Marburg, and to the disease as it here prevails, Dr. Falck has devoted especial attention. This village is situated in the valley of the Layñe, at a point where the hill, on the south-eastern slope of which it is situated, divides into branches following a south-westerly and easterly direction. The western hill is covered by forest; the northern by gardens belonging to the town of Marburg; and by these mountains the village, which lies partly on the slope, and partly in the valley, is protected from south and west winds. The south-west branch of the hill consists of the red sandstone beneath which is placed the "rothes Todtliegendes," with grauwacke and zechstein;—all the springs proceed from the red sandstone. The other branch of the hill consists of red sandstone broken in places by basalt. This valley, which forms the termination of that of the Layne, is divided into nearly equal portions by that river; and is chiefly devoted to the cultivation of corn, and to gardens. The river seldom overflows its banks; there are no marshes, and but little dry alluvial soil. The air of the valley is freely moved by winds, more especially those from the west, and the sun's rays are felt during the whole day in Ockershausen and Marburg.

The inhabitants of Ockershausen are subject to scrofulous affections as well as bronchocele. Intermittent fevers rarely if ever occur. The young persons are of the lymphatic temperament, and have yellow hair and blue or gray eyes; of 130 pupils in the village school, one only was found to have dark eyes. They are less intelligent than the children from the neighbouring villages. The adults are of large and robust forms; they labour idly, want useful vigour, and have a reputation for dishonesty in the neighbouring town. They generally attain an advanced age.

Bronchocele is most extensively diffused in this village. The infants have the thyroid gland larger than natural, and have the appearance described by Foderé as characteristic of the young persons in goitrous districts. Of the 130 children in the school, between the 6th and 14th year, 33 had bronchocele. The disease exists in all parts of the village, whether situated on elevated ground or in the valley. In 27 houses in one street, 38 goitrous persons were found; and of the whole of the inhabitants, amounting to 706, 180 were suffering from the disease. In size, the thyroid gland generally equalled that of a hen's or goose's egg, occasionally it was as large as the fist.

The disease appears usually to commence between the 10th and 20th years; of 80 children between 8 and 13 years of age, 24 had goitre; while of the remaining 50 between 6 and 7 years of age, 9 only were affected. In the Clinical Institution of Marburg, of 19 young persons treated from this village, the majority were between 16 and 18 years of age. Dr. Falck has thrice observed the disease congenital. As before remarked, in this village there does not appear to be any material difference between the liability to the disease in the two sexes; of the pupils in the school, 69 were boys and 61 girls, and of the former 17, and of the latter 16 were goitrous. Of the 80 children between 8 and 14 years of age, 41 were boys, and 39 girls, and of these, 13 and 11 respectively laboured under the disease; and of the remaining 50 children between 6 and 7 years of age, 28 were boys, and 22 girls, and 4 boys and 5 girls were goitrous. In males in more advanced life, the disease does not appear to make progress, but in females, (from the neck being exposed?) it continues to increase. Dr. Falck's observations confirm the assertion of Alibert, that the right lobe of the thyroid gland is that first affected; of the 33 goitrous children, 28 were found to have the right lobe enlarged, and in 5 the right and middle lobes were conjointly affected.

Dr. Falck remarks that no doubt can exist of bronchocele being endemic in Ockershausen. It affects those who in early life removed from the adjacent villages, and if those in advanced age are less susceptible to the operation of its causes, the disease certainly attacks their children. No decided evidence was afforded that the cattle in the district are liable to the disease.

The influence of hereditary predisposition in conducing to the development of goitre, though traceable, and chiefly on the maternal side, is less distinctly marked than is generally stated by authors. Dr. Falck is acquainted with families in which the mother, or both parents, have had bronchocele, yet where the children are wholly or partially free from the affection; and, on the other hand, he has known all the children of a family affected, where the parents were free. In several cases the affection of the thyroid gland coexisted with cretinism. In three girls he found imperfect development of the eye or its adjuncts, (1 microphthalmum, 2 ancyloblepharon;) and of these the mothers were goitrous. In another family in which bronchocele had existed for several generations, the father and all the children had a peculiar conformation of the nose, and marked mental hebetude. In two other families the mothers only were goitrous, but all the children were cretins.

The people live in huts made of wood, which, though inferior, are similar to those of the neighbouring peasantry; and there is no difference in the quality of food employed by the inhabitants of Ockershausen, from that of the population around, who, are wholly or partially, exempt from the disease.

The village is supplied with water by four wells; of these, two are situated near together, and the water proceeds from the red sandstone formation; a third from giving but little water is not generally employed, and the fourth, which is also situated in the red sandstone, yields only a scanty supply. The water from these wells is clear and limpid, and remains so during continued rain. It has the taste of distilled water. The temperature at the springs is 8·5 of Reaumur (51° Fahr.), that of the air being 5·5 Reaumur (44° Fahr.); and this does not vary with the season. Its specific gravity is 1·001; on analysis it indicated only traces of lime, magnesia, and sulphuric, muriatic, and carbonic acids. Free carbonic acid was entirely absent; 3742·41 grammes yielded on evaporation a scanty ponderable residue.

The inhabitants of the villages adjacent to Ockershausen, and situated some on strata of grauwacke and clay slate, others on the red sandstone, are very little affected by or entirely free from the disease; and this is also the case with the inhabitants of the village of Kappel, situated in the valley immediately below Ockershausen. Unfortunately the water employed as beverage in these villages was not analysed.

The conclusions at which Dr. Falck arrives from his observations in the two principalities are:

1. That though different authors have found bronchocele to be accurately confined in certain districts to particular strata of rocks, as in Kumaon, according to Maclelland, to transition limestone; in Wurtemburg, according to Riedle, to shell limestone; in England and Siberia, to magnesian limestone; in Hesse, to shell and magnesian limestone; in Switzerland to transition limestone and nagelflugh; yet that in several districts situated on similar formations, as in Wolfhagen and Hofgeismar, bronchocele rarely occurs.

2. The assertion of Maclelland that bronchocele is infrequent in districts where the primitive strata prevail, is opposed by the observations of Dr. Falck in Hesse. He has further been informed that bronchocele is frequent in the districts of Schierke, Lehrbach, and Neuwerk in the Hercynian forest, which are situated on primitive and transition rocks; and similar observations are recorded by Humboldt, De Vest, and Iphofen.

As, therefore, the disease cannot be regarded as prevailing only in situations where particular forms of rock abound—to what, asks Dr. Falck, can we ascribe its production? Can the absence of free carbonic acid gas from the water employed as beverage by the inhabitants of goitrous districts, afford a sufficient explanation of its development? That the origin of the disease is to be sought for in some noxious quality communicated to the water, either by the presence of some injurious ingredient, or the absence of a necessary element, is probable enough; how far, however, the absence of free carbonic acid gas may exert such influence can only be settled by observations collected in the several districts where the disease prevails. The observations of Dr. Falck, as to the quality of the water employed in the village of Ockershausen, and the previous notice of a similar kind by Iphofen,* are highly important; yet it is much to be regretted, that the former had not directed his attention to the chemical constituents of the water employed in the villages adjacent to Ockershausen, in which he informs us that bronchocele is little prevalent. We would add also, that his demonstration of the inapplicability of the conclusions of Maclelland as to the strata to which bronchocele is confined, would have been more satisfactory had he, in all the districts, informed us from what source the water employed as beverage by the inhabitants was derived; without this

* Der Cretinismus, Philos. und Medic. Untersucht. Dresden, 1817. 1 Th. § 77.

information we are left in doubt whether in any given district the inhabitants use the spring water of their own soil, or are supplied by rivers proceeding from strata of a totally different character.

Notwithstanding this defect, we regard the thesis of Dr. Falck as highly commendable. Our knowledge of the efficient cause or causes of bronchocele is extremely imperfect; nor can the history of any other subject in the wide field of experimental science, afford a more curious illustration of the varied and conflicting theories which may be deduced from the careless or too limited observation of facts; were, however, medical men in the different districts where the disease prevails, to devote themselves to record accurately the local peculiarities of climate, soil, the chemical qualities of the water, and all other circumstances which could exert any influence on its development,—a mass of facts would shortly be accumulated, which would afford the basis of satisfactory generalization. Observations of this kind have now been collected in several different districts where the disease is prevalent or endemic, and Dr. Falck has done much to supply this want in the countries, the illustration of which he has undertaken.

---

